# Emergence of Haldane Pseudo-Potentials in Systems with Short-Range Interactions

**DOI:** 10.1007/s10955-020-02586-0

**Published:** 2020-06-24

**Authors:** Robert Seiringer, Jakob Yngvason

**Affiliations:** 1 IST Austria, Am Campus 1, 3400 Klosterneuburg, Austria; 2 Faculty of Physics, University of Vienna, Boltzmanngasse 5, 1090 Vienna, Austria

## Abstract

In the setting of the fractional quantum Hall effect we study the effects of strong, repulsive two-body interaction potentials of short range. We prove that Haldane’s pseudo-potential operators, including their pre-factors, emerge as mathematically rigorous limits of such interactions when the range of the potential tends to zero while its strength tends to infinity. In a common approach the interaction potential is expanded in angular momentum eigenstates in the lowest Landau level, which amounts to taking the pre-factors to be the moments of the potential. Such a procedure is not appropriate for very strong interactions, however, in particular not in the case of hard spheres. We derive the formulas valid in the short-range case, which involve the scattering lengths of the interaction potential in different angular momentum channels rather than its moments. Our results hold for bosons and fermions alike and generalize previous results in [6], which apply to bosons in the lowest angular momentum channel. Our main theorem asserts the convergence in a norm-resolvent sense of the Hamiltonian on the whole Hilbert space, after appropriate energy scalings, to Hamiltonians with contact interactions in the lowest Landau level.

## Introduction

In a seminal paper [1] on the fractional quantum Hall effect^1^ F.D.M. Haldane introduced two-body interaction operators (pseudo-potentials) that have Laughlin’s wave functions [5] as exact eigenstates. In suitable units the latter are the functions

1.1 $$\begin{aligned} \Psi ^\mathrm{L}_{m} (x_1,\dots ,x_N) = C_{N,m} \prod _{i<j} (z_i-z_j)^{m} \prod _i \mathrm e^{-|x_i|^2/2} \end{aligned}$$

where $N\ge 2$ is the particle number, the $z_j=x^{(1)}_j+\mathrm i x^{(2)}_j\in \mathbb C$ with $x_j=(x^{(1)}_j, x^{(2)}_j)\in \mathbb R^2$ are complex coordinates for the particles moving in a two-dimensional plane perpendicular to a strong magnetic field, *m* is a positive integer (even for bosons, odd for fermions), and $C_{N,m}$ a normalization factor. The bosonic case, considered in [6] in the lowest angular momentum channel, is in particular relevant for dilute quantum gases in rapid rotation, where the rotational velocity takes the role of the magnetic field [7, 8].

Haldane’s pseudo-potential operators have the form

1.2 $$\begin{aligned} \sum _{i\ne j}\mathfrak P^{(\ell )}_{ij} \end{aligned}$$

where $\mathfrak P^{(\ell )}_{ij}$, for a nonnegative integer $\ell$, is the projector onto states in the lowest Landau level (LLL) with relative angular momentum $\ell$ for a pair *ij*. Recall that *N*-particle wave functions in the LLL are functions in $L^2(\mathbb R^{2N})$ of the form

1.3 $$\begin{aligned} \Psi (x_1,\dots ,x_N)=\psi (z_1,\dots , z_N)\prod _i \mathrm e^{-|x_i|^2/2} \end{aligned}$$

with analytic functions $\psi$. They are the lowest energy eigenfunctions of the magnetic kinetic energy part $H^{(0)}$ of the Hamiltonian (2.2) introduced below. Such a function has relative angular momentum $\ell$ with respect to a pair *ij* if

1.4 $$\begin{aligned} \psi (z_1,\dots , z_N)=(z_i-z_j)^\ell \varphi (z_1,\dots , z_N) \end{aligned}$$

with $\varphi$ depending on $z_i$ and $z_j$ only in the combination $z_i+z_j$. A general analytic function $\psi$ can be expanded in powers of the difference variable $z_i-z_j$ with $z_i+z_j$ and $z_k$, $k\ne i,j$, fixed, and $\mathfrak P^{(\ell )}_{i,j}$ picks out the $\ell$th derivative w.r.t. $(z_i-z_j)$ at zero, annihilating the other terms. Hence $\mathfrak P^{(\ell )}_{i,j}$ can be regarded as a zero-range interaction. In fact, as a quadratic form on states in the LLL with relative angular momentum $\ge \ell$, $\mathfrak P^{(\ell )}_{i,j}$ is formally equal to $2\pi (2^\ell \ell !)^{-1} \Delta ^{\ell } \delta (x_i-x_j)$ with $\Delta =\nabla ^2$ the Laplacian [9, 10]. A Laughlin wave function $\Psi ^\mathrm{L}_{m}$ is a zero energy ground state of (1.2) for all $m\ge \ell +1$.

The single-particle Hilbert space $L^{2}(\mathbb R^2)$ splits into Landau levels corresponding to the eigenvalues 4*n*, $n=0,1,\dots$ of the single particle magnetic kinetic energy (2.1). The full *N*-particle Hilbert space $\mathcal H=L^2(\mathbb R^{2N})$ (or its antisymmetric or symmetric part, for fermions or bosons respectively) is a direct sum of the space where all particles are in the lowest Landau level, denoted LLL as above, and its orthogonal complement, where some particles are in higher Landau levels.

Contrary to (1.2), *bona-fide* potentials, defined as multiplication operators by measurable functions of $x\in \mathbb R^2$, do not leave the LLL invariant but generate also states in higher Landau levels. In [1] the pseudo-potentials were obtained by expanding the projection onto the lowest Landau level of a radial interaction potential $v(x_i-x_j)$ into angular momentum eigenstates, i.e, writing

1.5 $$\begin{aligned} P_\mathrm{LLL} v(x_i-x_j)P_\mathrm{LLL}=\sum _{\ell \ge 0}\langle \varphi _\ell |v|\varphi _\ell \rangle \mathfrak P^{(\ell )}_{i,j} \end{aligned}$$

where $P_\mathrm{LLL}$ is the projector onto the LLL (in all variables) and

1.6 $$\begin{aligned} \varphi _\ell (z)=(\pi \ell !)^{-1/2} z^\ell \mathrm e^{-|z|^2/2} \end{aligned}$$

the single-particle angular momentum eigenfunctions in the lowest Landau level.^2^ In the simplest case, $\ell =0$, the expansion coefficient is essentially the integral $\int v$ (except for a Gaussian factor), and for higher $\ell$ it is proportional to the $2\ell$th moment, $\int r^{2\ell }\,v$. It is, however, clear that this is not viable for very strong interaction potentials, in particular not if *v* has a genuine hard core so that all its moments are infinite. In order for (1.5) to be meaningful it is necessary that the quadratic form domain of the multiplication operator $v(x_i-x_j)$ has a nontrivial intersection with the LLL.

To obtain valid formulas in the limit when the range of the interaction tends to zero but its strength to infinity, it is necessary to take the kinetic energy operator in higher Landau levels into account. Indeed, the local structure of wave functions in the LLL is quite restricted due to analyticity. Wave functions with finer structure, avoiding configurations where a *bona fide* interaction potential is very large, must necessarily have components also in higher Landau levels. Although these components may tend to zero as the range tends to zero, the joint effects of the kinetic and potential energies at length scales much smaller than the magnetic length^3^ will leave a trace in the LLL, and lead in particular to a replacement of $\int v$ as the coupling constant in front of (1.2) by a constant proportional to the *s*-wave scattering length of *v*, as rigorously established in [6].

In the present paper we generalize the analysis in [6] to include all angular momentum channels. This allows in particular also to treat fermions, where only odd angular momenta occur. To account for the fact that strong, short range interactions may create states with arbitrarily large energy in ever higher Landau levels it is convenient to consider convergence of operators in resolvent sense. An elegant way to achieve this uses the concept of $\Gamma$*-convergence* [13] which involves resolvents of operators. The resolvents of Hamiltonians with strong interaction suppress states with very high energy and eventually, in the limit considered, only states in the LLL survive.

Our main theorem states that after suitable $\ell$-dependent energy scaling the full Hamiltonian converges in this sense to a Hamiltonian in the lowest Landau level with the pseudo-potential interaction operator (1.2) and a definite coupling constant. Generalizing the $\ell =0$ case, the coupling constant is determined by the $\ell$-wave scattering length of *v*, which can be obtained from a variational principle. It is essential here to note that the effective Hamiltonian in the LLL is *not* obtained by projecting the original Hamiltonian onto the LLL. The effective coupling constants are renormalized by properly taking into account the behavior of the system at length scales much shorter than the magnetic length. In the limit of zero range/infinite strength one obtains a Hamiltonian that operates within the LLL with the renormalized coupling constants.

In contrast to [6] we shall mainly focus on strictly two-dimensional systems for simplicity, but in Sect. 4 we discuss the modifications that are necessary to apply our results to three-dimensional systems with a confining potential in the third direction. To keep the proofs simple and transparent, we do not keep track of the *N*-dependence of our estimates but rather regard the particle number as fixed. Quantitative, *N*-dependent estimates as derived in [6] for $\ell =0$ would also be possible with some additional work. The estimates in [6] are far from optimal, however, and we leave it as an open problem to improve these estimates and generalize them to $\ell \ge 1$.

Our results establish the Laughlin functions (1.1) as exact ground states of magnetic Hamiltonians with interactions through a rigorous limit procedure. We emphasize, however, that this procedure requires a strong interaction potential of short range. In theoretical studies of Bose-Einstein condensation in dilute atomic gases [11, 12], the interactions between atoms and molecules are often modeled by strong repulsive potentials, even as hard spheres, of range much smaller than the mean particle distance. Such models are prime examples to which our results apply, the $\ell =0$ case for bosons treated in [6] being the simplest one. The role of the magnetic field may here be taken over by the angular velocity of a system in rapid rotation [7, 8].

In quantum Hall physics for electrons, on the other hand, the dominant interaction is the Coulomb interaction between the particles (in addition to external potentials modeling traps and impurities) and is thus of a different character than in the examples just mentioned. Numerical studies indicate that Laughlin states may have large overlap with true ground states in quantum Hall settings (see, e.g., [2], Sect. 8.7), and, in fact, Haldane’s expansion (1.5) applied to a Coulomb potential produces terms that decrease rapidly with increasing $\ell$. So far, no rigorous justification for the pseudo-potential description in the Coulomb case is known, however.

## Model and Main Results

On $L^2(\mathbb {R}^2)$, define the operator $h\ge 0$ as

2.1 $$\begin{aligned} h = ( -\mathrm i\nabla - x^\perp )^2 - 2 \end{aligned}$$

where $x^\perp = (-x^{(2)},x^{(1)})$ for $x=(x^{(1)},x^{(2)})\in \mathbb {R}^2$. It is simply the magnetic Laplacian for magnetic field $B=2$, with its ground state energy shifted to zero, having spectrum $4\mathbb {N}\cup \{0\}$. The particle mass has been set to 1/2 and Planck’s constant to 1. For $v\ge 0$ radial and of compact support, and $N\ge 2$, define the *N*-particle Hamiltonian

2.2 $$\begin{aligned} H_a = H^{(0)}+ \sum _{1\le i<j\le N} v_a ( |x_i-x_j| ) \end{aligned}$$

on $\mathcal {H}= L^2(\mathbb {R}^{2N})$, where $H^{(0)}=\sum _{i=1}^N h_i$ is the *N*-particle magnetic kinetic energy operator and

2.3 $$\begin{aligned} v_a(r) = a^{-2} v(r/a) \end{aligned}$$

for $a>0$. This scaling appears naturally in our choice of units, reflecting the fact that $-\nabla ^2$ scales like the square of an inverse length. For short-range interactions, the scattering length is the natural parameter measuring their strength, and the scattering length of $v_a$ equals *a* times the one of *v*. We are interested in the regime where *a* is much smaller than the magnetic length, which is *O*(1) in our units, i.e., we consider the case $a\ll 1$. In particular, the strength $a^{-2}$ of the interaction is much larger than the energy gap between Landau levels.^4^

We assume that *v* is a non-negative measurable function of compact support, but we don’t need to assume that *v* is integrable; in particular, it is allowed to have a hard core, i.e., be infinite on a set of positive measure. Functions in the domain of the Hamiltonian then vanish on the corresponding set in configuration space. It is not necessary to restrict to symmetric or anti-symmetric functions, our analysis is valid on the whole Hilbert space $\mathcal {H}= L^2(\mathbb {R}^{2N})$ and applies equally to bosons and fermions.

We introduce the sequence of closed subspaces

2.4 $$\begin{aligned} \mathcal {H}\supset \mathfrak {B}_0 \supset \mathfrak {B}_1 \supset \dots \end{aligned}$$

where $\mathfrak {B}_\ell$ for $\ell \ge 0$ consists of $\Psi \in \mathcal {H}$ of the form

2.5 $$\begin{aligned} \Psi (x_1,\dots ,x_N) = \mathrm e^{-\tfrac{1}{2} \sum _{i=1}^N |x_i|^2} \varphi (z_1,\dots ,z_N) \prod _{i<j} (z_i - z_j)^\ell \end{aligned}$$

with $\varphi : \mathbb {C}^N\rightarrow \mathbb {C}$ analytic. Here we identify $z_j \in \mathbb {C}$ with $x_j = (x^{(1)}_j, x^{(2)}_j) \in \mathbb {R}^2$ via $z_j = x^{(1)}_j + \mathrm ix^{(2)}_j$. Note that $\mathfrak {B}_0$ coincides with the LLL, i.e., the kernel of $H^{(0)}$. We also note that for large *N* all normalized wave functions in $\mathfrak {B}_\ell$ have a remarkable incompressibility property: their one-particle density, suitably averaged, is everywhere bounded above by $(\pi \ell )^{-1}$ [14–16].

On the space $\mathfrak {B}_\ell$, we define the operators

2.6 $$\begin{aligned} \mathfrak {h}_\ell = \sum _{i<j} \mathfrak {D}^{(\ell )}_{ij} \end{aligned}$$

where $\mathfrak {D}^{(\ell )}$ is for $\ell \ge 0$ defined via the quadratic form for a two-particle wave function $\Psi (x_1,x_2) = \mathrm e^{-\tfrac{1}{2} (|x_1|^2+|x_2|^2)} \varphi (z_1,z_2) (z_1-z_2)^\ell$ as

2.7 $$\begin{aligned} \langle \Psi | \mathfrak {D}^{(\ell )} \Psi \rangle = \int _{\mathbb {R}^2} \mathrm e^{-2|x|^2} | \varphi (z,z)|^2 dx \end{aligned}$$

and $\mathfrak {D}^{(\ell )}_{ij}$ acts on an *N*-particle wave function in $\mathfrak {B}_\ell$ like $\mathfrak {D}^{(\ell )}$ w.r.t. the variables $z_i$, $z_j$ if the others are fixed. Note that $\mathfrak {D}^{(\ell )}_{ij}$ is a bounded operator; in fact, by comparing expectation values and using (1.6), one sees that

2.8 $$\begin{aligned} \mathfrak {D}^{(\ell )}_{ij}=(\pi \ell !)^{-1}\mathfrak P_{ij}^{(\ell )} \end{aligned}$$

on $\mathfrak {B}_\ell$, with the previously introduced projection $\mathfrak P_{ij}^{(\ell )}$ on states with relative angular momentum $\ell$ for a pair *ij*. Hence $\mathfrak {h}_\ell$ is equal to (1.2) on $\mathfrak {B}_\ell$, up to the factor $(\pi \ell !)^{-1}$. Moreover, the kernel of $\mathfrak {h}_\ell$ coincides with $\mathfrak {B}_{\ell +1}$, the domain of $\mathfrak {h}_{\ell +1}$.

Next we define the relevant scattering parameters in arbitrary angular momentum channels, generalizing the approach to the *s*-wave scattering length in [12, App. C] and [17]. For $\ell \in \mathbb {N}$, $\ell \ge 1$, define $b_\ell$ via the variational principle

2.9 $$\begin{aligned} b_\ell = \frac{1}{4 \pi \ell } \min \left\{ \int _{\mathbb {R}^2} |x|^{2\ell } \left( |\nabla f(x)|^2 + \tfrac{1}{2} v(|x|) |f(x)|^2 \right) dx \, : \, \lim _{|x|\rightarrow \infty } f(x) = 1\right\} . \end{aligned}$$

We denote the minimizer by $f_\ell$. It is radially symmetric, satisfies $0\le f_\ell \le 1$, and $f_\ell (|x|) = 1 - b_\ell /|x|^{2\ell }$ for *x* outside the support of *v*, i.e., $|x|> R_0$. In particular, $b_\ell = R_0^{2\ell }$ for hard discs. The variational equation (zero-energy scattering equation in the $\ell$ channel) for the minimizer reads, with $r=|x|$,

2.10 $$\begin{aligned} - f_\ell ^{\prime \prime }(r)-(2\ell +1)r^{-1}f_\ell ^\prime (r)+\frac{1}{2}v(r)f_\ell (r)=0. \end{aligned}$$

For $\ell =0$ the zero energy scattering solution is not bounded but rather grows logarithmically at infinity. We take $R>R_0$ and define the *s*-wave scattering length $b_0$ as in [17] by

2.11 $$\begin{aligned} \frac{4\pi }{\ln (R^2/b_0^2)} = \min \left\{ \int _{\mathbb {R}^2} \left( |\nabla f(x)|^2 + \tfrac{1}{2} v(|x|) |f(x)|^2 \right) dx \, : \, f(x) = 1 \hbox { for } |x|\ge R\right\} . \end{aligned}$$

As shown in [17], $b_0$ is independent of *R*; for hard discs $b_0=R_0$.

If *v* is replaced by $v_a$ then, by a change of variables in the integrals (2.9) and (2.11), one sees that $b_\ell$ is replaced by $a^{2\ell }b_\ell$ for $\ell \ge 1$ and $b_0$ by $ab_0$.

With these definitions, our main result can be formulated as follows.

### Theorem 1

For any $\ell \ge 1$, the operator $a^{-2\ell } H_a$ converges to $8\pi \ell b_\ell \mathfrak {h}_\ell$ in strong resolvent sense as $a\rightarrow 0$, i.e., for any $\mu > 0$ and $\psi \in \mathcal {H}= L^2(\mathbb {R}^{2N})$

2.12 $$\begin{aligned} \lim _{a\rightarrow 0} (\mu + a^{-2\ell } H_a )^{-1} \psi = (\mu + 8\pi \ell b_\ell \mathfrak {h}_\ell )^{-1} P_\ell \psi \end{aligned}$$

strongly in $L^2(\mathbb {R}^{2N})$, where $P_\ell$ denotes the projection onto $\mathfrak {B}_\ell \subset \mathcal {H}$. Moreover, for $\ell =0$, $\ln (1/a^2) H_a$ converges in the same sense to $8\pi \mathfrak {h}_0$.

### Remark 1

In Theorem 1 we assume that the interaction potential *v* is not identically zero. If it is, we have $b_\ell = 0$ for all $\ell$, and (2.12) trivially holds with $P_\ell$ replaced by $P_0$ in this case.

### Remark 2

If one introduces a coupling parameter, i.e., replaces *v* by $\lambda v$ for $\lambda >0$, one can explore, in addtion, the regimes of weak and strong coupling. In case *v* is suitably regular and $\lambda \ll 1$, the parameters $b_\ell$ are to leading order given by their Born approximation, $b_\ell \approx (8\pi \ell )^{-1} \lambda \int r^{2\ell } v$, corresponding to the choice $f\equiv 1$ in (2.9). That is, for weak potentials, one recovers the moments of *v* as the pre-factors of the pseudo-potentials, as predicted by first-order perturbation theory. In the limit of strong coupling $\lambda \gg 1$, on the other hand, one obtains the scattering parameters of hard spheres, solely determined by their diameter.

**Fig. 1:**
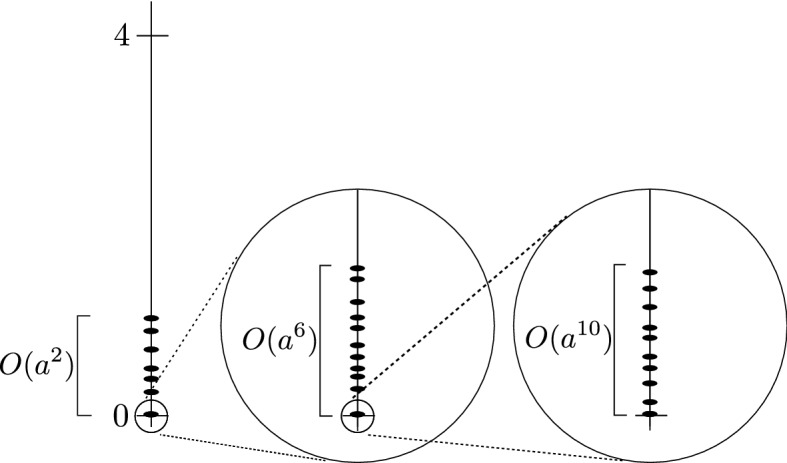
Sketch of the spectrum in the fermionic case. States with energy of order $a^2$ are described by the effective Hamiltonian $\mathfrak {h}_{1}$ on the LLL. Zooming in on its kernel, one finds $\mathfrak {h}_{3}$ as an effective Hamiltonian, describing states with energy of order $a^6$, and so forth

The result in Theorem 1 can be interpreted as follows. States in $\mathcal {H}$ with energy of order $a^{2\ell }$ are, for small *a*, necessarily close to states in $\mathfrak {B}_\ell$, and are described by an effective Hamiltonian $\mathfrak {h}_\ell$. On the kernel of $\mathfrak {h}_\ell$, one can zoom in further by looking at energies of order $a^{2\ell '}$ for some $\ell '>\ell$, and find a new effective Hamiltonian $\mathfrak {h}_{\ell '}$. In the limit $a\rightarrow 0$, one thus obtains an infinite cascade of effective Hamiltonians in the corresponding energy windows. See Fig. 1 for an illustration.

Theorem 1 readily implies that, for any hermitian bounded operator *K* on $\mathcal {H}$ that is independent of *a*,

2.13 $$\begin{aligned} a^{-2\ell } H_a + K \ \xrightarrow {a\rightarrow 0} \ 8\pi \ell b_\ell \mathfrak {h}_\ell + P_\ell K P_\ell \end{aligned}$$

in strong resolvent sense, and this generalizes to suitable unbounded *K*. One could consider an additional interaction potential, for instance of Coulomb type, in which case $P_\ell K P_\ell$ is a linear combination of the operators $\sum _{i\ne j}\mathfrak P^{(\ell ')}_{ij}$ as in (1.5), but with the sum restricted to angular momenta $\ell '\ge \ell$. If one adds a confining potential, it is not difficult to show that the convergence in (2.13) holds actually in the norm-resolvent sense, i.e., the operator norm of the difference between the resolvents of the left and right side of (2.13) tends to zero as $a\rightarrow 0$. In fact, the resolvents are in this case compact operators and $( \mu + a^{-2\ell } H_a +K)^{-1} \le (\mu + H^{(0)} + K)^{-1}$ for any $a\le 1$ and $\mu > - \hbox {inf\, spec\,} K$, where we used the assumption that *v* is non-negative. One readily checks that strong- and norm-resolvent convergence are equivalent for a sequence of non-negative operators that are dominated by a fixed compact operator.

A convenient choice is the harmonic oscillator potential $K= \sum _{i=1}^N |x_i|^2$, which acts as $P_\ell K P_\ell = (N+L)P_\ell$, with $L = \sum _{i=1}^N (z_i \partial _{z_i}-\bar{z}_i \partial _{\bar{z}_i}) = \sum _{i=1}^N z_i \partial _{z_i}$ the total angular momentum operator in the LLL. Indeed, $\ell \delta _{\ell m}=\langle \varphi _\ell , L \varphi _m\rangle =\langle \varphi _\ell ,(|z|^2-1)\varphi _m\rangle$ using (1.6). In particular, for any $\lambda >0$ and $\ell \ge 1$ we have

2.14 $$\begin{aligned} a^{-2\ell } H_a + \lambda \sum _{i=1}^N |x_i|^2 \ \xrightarrow {a\rightarrow 0} \ 8\pi \ell b_\ell \mathfrak {h}_\ell + \lambda (N+L)P_\ell \end{aligned}$$

in *norm*-resolvent sense. This extends to $\ell =0$ in the same way as explained in Theorem 1.

The right side of (2.14) is the sum of two commuting operators on $\mathfrak {B}_\ell$, which we write for short as

2.15 $$\begin{aligned} \gamma \mathfrak {h}_\ell +\lambda \mathcal L \end{aligned}$$

with $\mathcal L=(N+L)P_\ell$ and $\gamma ,\lambda >0$, and we consider the joint spectrum of the commuting operators $\mathfrak {h}_\ell$ and $\mathcal L$. For a fixed eigenvalue of $\mathcal L$ we have a finite dimensional space on which $\mathfrak {h}_\ell$ has nonnegative eigenvalues, and the situation is analogous to the discussion of the *Yrast curve* (see [6, Fig. 1] for a sketch). This curve is the (convex hull of the) boundary of the joint spectrum. By varying the ratio $\lambda /\gamma$ we can adjust the value(s) of the angular momentum where the energy is minimal and thereby change the ground state(s) of (2.15). Indeed, the ground state is determined by the point(s) where a line with slope $-\lambda /\gamma$ touches the joint spectrum. For fixed $\gamma$ and $\lambda$ small enough (depending on the spectral gap of $\mathfrak {h}_\ell$) the unique ground state equals the Laughlin state $\Psi ^\mathrm{L}_{\ell +1}$, where $\mathfrak {h}_\ell$ has eigenvalue 0. It is separated from other states with the same or less angular momentum by a spectral gap since the state space is finite dimensional, but the size of the gap might *a-priori* depend on the particle number *N*. It is an important, but still unproved, conjecture in FQHE physics that this gap has a strictly positive lower bound independent of *N* (see, e.g., [16]). For $\lambda$ large enough the unique ground state of (2.15) is the Laughlin wave function $\Psi ^\mathrm{L}_\ell$, where $\mathfrak {h}_\ell$ is strictly positive but $\mathcal L$ takes its minimal value in $\mathfrak {B}_\ell$.

The norm-resolvent convergence in (2.14) implies convergence of eigenvalues and corresponding eigenvectors, and hence we can conclude the following corollary:

### Corollary 1

Fix $\ell \ge 1$. For $\lambda >0$ small enough, the ground state of $H_a + a^{2\ell } \lambda \sum _{i=1}^N |x_i|^2$ converges in $L^2(\mathbb {R}^{2N})$ to $\Psi ^\mathrm{L}_{\ell +1}$ as $a\rightarrow 0$. For $\ell =0$ this holds with $a^{2\ell }$ replaced by $(\ln (1/a^2))^{-1}$.

These results hold on the whole Hilbert space $\mathcal {H}=L^2(\mathbb {R}^{2N})$ without symmetry constraints. When restricting to the bosonic and fermionic subspaces, respectively, we have $\mathfrak {B}_{\ell -1} = \mathfrak {B}_{\ell }$ for $\ell$ even (bosons) or $\ell$ odd (fermions), hence it is natural to restrict to such $\ell$ depending on the symmetry. The unique ground state of the right side of (2.14) is now $\Psi ^\mathrm{L}_{\ell +2}$ for small $\lambda$. In particular, Corollary 1 holds with $\Psi ^\mathrm{L}_{\ell +1}$ replaced by $\Psi ^\mathrm{L}_{\ell +2}$ in the bosonic subspace for even $\ell$, and in the fermionic subspace for odd $\ell$.

### Remark 3

Our results can be extended to a system in three spatial dimensions with an additional confinement in the third direction. This will be detailed in Sect. 4, thereby generalizing a corresponding result for the special case $\ell =0$ in [6].

## Proof of Theorem 1

### Gamma Convergence

It is well known that strong resolvent convergence of operators is equivalent to $\Gamma$-convergence of the corresponding quadratic forms in both weak and strong topologies (see [13, Sec. 13]). Hence we find it convenient to reformulate Theorem 1 in terms of $\Gamma$-convergence.

We define the functions $F_a : \mathcal {H}\rightarrow [0,\infty ]$ as

3.1 $$\begin{aligned} F_a(\Psi ) = \Vert \sqrt{H_a} \Psi \Vert ^2 \end{aligned}$$

(with the understanding that $F_a$ equals $+\infty$ if $\Psi$ is not in the form domain of $H_a$). Moreover, for $\ell \ge 0$, define $G^{(\ell )}:\mathcal {H}\rightarrow [0,+\infty ]$ as

3.2 $$\begin{aligned} G^{(\ell )}(\Psi ) = {\left\{ \begin{array}{ll} \langle \Psi | \mathfrak {h}_\ell \Psi \rangle &{} \text {if}\ \Psi \in \mathfrak {B}_\ell \\ +\infty &{} \text {otherwise.} \end{array}\right. } \end{aligned}$$

Theorem 1 is an immediate consequence of the following Proposition.

#### Proposition 1

For any $$\ell \ge 1$$, the function $$a^{-2\ell } F_a$$$$\Gamma $$-converges to $$8\pi \ell b_\ell G^{(\ell )}$$ as $$a\rightarrow 0$$ both in the strong and weak topology on $$\mathcal {H}$$. That is, for $$\ell \ge 1$$,

1. for any sequence $\{a_n\}_{n\in \mathbb {N}}$ of positive numbers with $\lim _{n\rightarrow \infty } a_n = 0$, and any sequence $\{\Psi _n\}_{n\in \mathbb {N}}$ in $\mathcal {H}$ with $\Psi _n \rightharpoonup \Psi \in \mathcal {H}$ as $n\rightarrow \infty$,
  3.3 $$\begin{aligned} \liminf _{n\rightarrow \infty } a_n^{-2\ell } F_{a_n} ( \Psi _n) \ge 8\pi \ell b_\ell G^{(\ell )}(\Psi )\,. \end{aligned}$$
2. for any $\Psi \in \mathcal {H}$ and any sequence $\{a_n\}_{n\in \mathbb {N}}$ of positive numbers with $\lim _{n\rightarrow \infty } a_n = 0$, there exists a sequence $\{\Psi _n\}_{n\in \mathbb {N}}$ in $\mathcal {H}$ with $\Psi _n \rightarrow \Psi \in \mathcal {H}$ as $n\rightarrow \infty$ such that
  3.4 $$\begin{aligned} \lim _{n\rightarrow \infty } a_n^{-2\ell } F_{a_n} ( \Psi _n) = 8\pi \ell b_\ell G^{(\ell )}(\Psi )\,. \end{aligned}$$

For $\ell =0$, the same holds with $a^{-2\ell }$ replaced by $\ln (1/a^2)$, and $\ell b_\ell$ replaced by 1.

Note that (3.3) holds for all weakly converging sequences, while the convergence in (2) is strong, hence indeed there is $\Gamma$-convergence in both topologies. The proof of Proposition 1 will be given in the remainder of this section. We discuss the case $\ell \ge 1$ in detail, and indicate the modifications for $\ell =0$ in the last Subsect. 3.5.

### Dyson Lemma

We start with the following *Dyson Lemma*, so named because Dyson proved a first estimate of this type in 1957 [18] to obtain a lower bound on the energy of a hard core Bose gas. Subsequently this was extended in several ways, see [17, 19, 20] and, for the present context, in particular [6]. Lemma 4 in [6] is stated and proved for $\ell =0$, but since in that paper only the three-dimensional case was considered, we shall need an extra discussion for $\ell =0$ here, cf. Sec. 3.5.

We again identify $x=(x^{(1)},x^{(2)}) \in \mathbb {R}^2$ with $z = x^{(1)} + \mathrm ix^{(2)} \in \mathbb {C}$, and write $\partial _{\bar{z}} = \frac{1}{2}( \partial _{x^{(1)}} +\mathrm i \partial _{x^{(2)}})$, $\partial _{z} = \frac{1}{2}(\partial _{x^{(1)}} - \mathrm i\partial _{x^{(2)}})$.

#### Lemma 1

For any $\ell \ge 1$ and $R>a R_0$, and any $y\in \mathbb {C}$, we have

3.5 $$\begin{aligned}&\int _{|z-y| < R} \mathrm e^{-|z|^2} \left( 4 |\partial _{\bar{z}} \varphi (x)|^2 + \tfrac{1}{2} v_a(|z-y|) |\varphi (x)|^2 \right) dx \nonumber \\&\quad \ge 4\pi \ell b_\ell a^{2\ell } \mathrm e^{|y|^2 -R^2 } \left| \frac{1}{2\pi \mathrm i} \oint _{|z-y|=R} \mathrm e^{-z \bar{y}}\frac{ \varphi (x)}{(z-y)^{\ell +1}} dz \right| ^2 \end{aligned}$$

where *dz* stands for the complex line element.

Note that if $\varphi$ is analytic and vanishes like $\kappa (z-y)^{\ell }$ as $z\rightarrow y$ for some $\kappa \in \mathbb {C}$, the right side is proportional to $|\kappa |^2 \mathrm e^{ -|y|^2 }$.

#### Proof

For $R>a R_0$, consider the expression

3.6 $$\begin{aligned} A = \int _{|z|<R} \bar{z}^\ell \left[ 4 \left( \partial _z f(x) \right) \left( \partial _{\bar{z}} \varphi (x) \right) + \tfrac{1}{2} v_a(|z|) f(x) \varphi (x) \right] dx \end{aligned}$$

where $f(x) = f_\ell (x/a)$ with $f_\ell$ the minimizer in (2.9). The Cauchy–Schwarz inequality and the fact that $|\partial _z f| = \frac{1}{2} |\nabla f|$ (since *f* is real) imply that

3.7 $$\begin{aligned} |A|^2&\le \int _{|x|< R} |x|^{2\ell } \left( |\nabla f(x)|^2 + \tfrac{1}{2} v_a( |x|) |f (x)|^2 \right) dx \nonumber \\&\quad \times \int _{|x| < R} \left( 4 |\partial _{\bar{z}} \varphi (x)|^2 + \tfrac{1}{2} v_a(|x|) |\varphi (x)|^2 \right) dx. \end{aligned}$$

The term in the first line is bounded above by $4\pi \ell b_\ell a^{2\ell }$, hence

3.8 $$\begin{aligned} \int _{|x| < R} \left( 4 |\partial _{\bar{z}} \varphi (x)|^2 + \tfrac{1}{2} v_a(|x|) |\varphi (x)|^2 \right) dx \ge \frac{|A|^2}{4\pi \ell b_\ell a^{2\ell }}. \end{aligned}$$

Integrating by parts and using the variational equation (2.10) for *f* (and the fact that *f* is radial), we also have

3.9 $$\begin{aligned} A&= \int _{|z|<R} |z|^{2\ell } \left[ 4 \left( \partial _z f(x) \right) \left( \partial _{\bar{z}} z^{-\ell }\varphi (x) \right) +\tfrac{1}{2} v_a( |z|) f(x) z^{-\ell } \varphi (x) \right] dx \nonumber \\&= 4\pi \ell b_\ell a^{2\ell } \frac{1}{2\pi \mathrm i} \oint _{|z|=R} \frac{ \varphi (x)}{z^{\ell +1}} dz. \end{aligned}$$

We conclude that

3.10 $$\begin{aligned} \int _{|x| < R} \left( 4 |\partial _{\bar{z}} \varphi (x)|^2 + \tfrac{1}{2} v_a(|x|) |\varphi (x)|^2 \right) dx \ge 4\pi \ell b_\ell a^{2 \ell } \left| \frac{1}{2\pi \mathrm i} \oint _{|z|=R} \frac{ \varphi (x)}{z^{\ell +1}} dz \right| ^2. \end{aligned}$$

To bring this into the desired form, we bound for $y\in \mathbb {C}$

3.11 $$\begin{aligned}&\int _{|x|< R} \mathrm e^{-|z+y|^2} \left( 4 |\partial _{\bar{z}} \varphi (x)|^2 + \tfrac{1}{2} v_a(|x|) |\varphi (x)|^2 \right) dx \nonumber \\&\quad = \mathrm e^{-|y|^2} \int _{|x|< R} \mathrm e^{-|z|^2} \left( 4 |\partial _{\bar{z}} \mathrm e^{-z \bar{y}}\varphi (x)|^2 +\tfrac{1}{2} v_a( |x|) | \mathrm e^{-z \bar{y}}\varphi (x)|^2 \right) dx \nonumber \\&\quad \ge \mathrm e^{-|y|^2 -R^2 } \int _{|x| < R} \left( 4 |\partial _{\bar{z}} \mathrm e^{-z \bar{y}}\varphi (x)|^2 +\tfrac{1}{2} v_a( |x|) | \mathrm e^{-z \bar{y}}\varphi (x)|^2 \right) dx \nonumber \\&\quad \ge 4\pi \ell b_\ell a^{2\ell } \mathrm e^{-|y|^2 -R^2 } \left| \frac{1}{2\pi \mathrm i} \oint _{|z|=R} \mathrm e^{-z \bar{y}}\frac{ \varphi (x)}{z^{\ell +1}} dz \right| ^2. \end{aligned}$$

In particular, changing variables from *z* to $z-y$, Eq. (3.5) follows.

By averaging the bound (3.5) over *R*, we obtain as an immediate corollary

3.12 $$\begin{aligned}&\int _{|z-y| < R} \mathrm e^{-|z|^2} \left( 4 |\partial _{\bar{z}} \varphi (x)|^2 + \tfrac{1}{2} v_a(|z-y|) |\varphi (x)|^2 \right) dx \nonumber \\&\quad \ge 4\pi \ell b_\ell a^{2\ell } \mathrm e^{|y|^2 } \int _0^\infty \mathrm e^{-r^2} \rho (r) \left| \frac{1}{2\pi \mathrm i} \oint _{|z-y|=r} \mathrm e^{-z \bar{y}}\frac{ \varphi (x)}{(z-y)^{\ell +1}} dz \right| ^2 dr \end{aligned}$$

for any non-negative function $\rho$ supported on $[a R_0, R]$ with $\int \rho = 1$. We shall choose $\rho$ bounded, supported on [*R*/2, *R*], and independent of *a*; an explicit choice is $\rho (r)=2/R$ for $R/2<r<R$, and 0 otherwise. As long as $a < R/(2R_0)$, (3.12) holds for this choice of $\rho$.

### Lower Bound

We now turn to the proof of part (1) of Prop. 1 and establish the lower bound (3.3). We start by noting that, for any $\Psi \in L^2(\mathbb {R}^2)$ of the form $\Psi (x) = \mathrm e^{-|x|^2/2} \varphi (x)$, we have the representation

3.13 $$\begin{aligned} \langle \Psi | h \Psi \rangle = 4\int _{\mathbb {R}^2} \mathrm e^{-|x|^2} \left| \partial _{\bar{z}} \varphi (x) \right| ^2 dx \end{aligned}$$

where we denote $\partial _{\bar{z}} =\frac{1}{2}( \partial _{x^{(1)}} + \mathrm i\partial _{x^{(2)}})$ as above. This representation, in combination with (3.12), implies the lower bound

3.14 $$\begin{aligned} a^{-2\ell } F_a(\Psi )&\ge 4\pi \ell b_\ell \sum _{i \ne j}^N \int _{\mathbb {R}^{2(N-1)}} \mathrm e^{- \sum _{k, k\ne i,j }^N |x_k|^2} \chi _{i,R}(x_1,\dots , \, \not \! x_i, \dots , x_N) \nonumber \\&\quad \times \int _0^\infty \mathrm e^{-r^2} \rho (r) \left| \frac{1}{2\pi \mathrm i} \oint _{|z_i-z_j|=r} \mathrm e^{-z_i \bar{z}_j }\frac{ \varphi (x_1,\dots ,x_N)}{(z_i-z_j)^{\ell +1}} dz_i \right| ^2 dr\, \prod _{j, j\ne i}^N dx_j \end{aligned}$$

for $$\Psi (x_1,\dots ,x_N) = \mathrm e^{-\tfrac{1}{2} \sum _{i=1}^N |x_i|^2} \varphi (x_1,\dots ,x_N)$$, where

3.15 $$\begin{aligned} \chi _{i,R}(x_1,\dots , \, \not \! x_i, \dots , x_N)= \prod _{j<k, j,k\ne i} \theta ( |x_j - x_k| - 2R) \end{aligned}$$

restricts the integration to the set where $|x_j-x_k|\ge 2R$ for all $j,k\ne i$.

We claim that the quadratic form defined on the right side of (3.14) is bounded. In fact, by a simple Cauchy–Schwarz inequality, using that $\mathfrak {R}z_i \bar{z}_j = \frac{1}{2} (|z_i|^2 + |z_j|^2- |z_i-z_j|^2 )$,

3.16 $$\begin{aligned}&\left| \frac{1}{2\pi \mathrm i} \oint _{|z_i-z_j|=r} \mathrm e^{-z_i \bar{z}_j }\frac{ \varphi (x_1,\dots ,x_N)}{(z_i-z_j)^{\ell +1}} dz_i \right| ^2 \nonumber \\&\quad \le \frac{ \mathrm e^{r^2} }{2\pi r^{2\ell +1} } \oint _{|z_i-z_j|=r} \mathrm e^{-|x_i|^2 - |x_j|^2} |\varphi (x_1,\dots ,x_N)|^2 | dz_i| \,, \end{aligned}$$

which after integration over *r* and $x_j$ implies that

3.17 $$\begin{aligned}&\int _0^\infty \mathrm e^{-r^2} \rho (r) \int _{\mathbb {R}^2} \left| \frac{1}{2\pi \mathrm i} \oint _{|z_i-z_j|=r} \mathrm e^{-z_i \bar{z}_j }\frac{ \varphi (x_1,\dots ,x_N)}{(z_i-z_j)^{\ell +1}} dz_i \right| ^2 dr \, dx_j \nonumber \\&\quad \le \frac{1}{2\pi } \int _{\mathbb {R}^4} \mathrm e^{-|x_i|^2 - |x_j|^2} |\varphi (x_1,\dots ,x_N)|^2 \frac{\rho (|x_i-x_j|)}{|x_i - x_j|^{2\ell +1}} dx_i \, dx_j. \end{aligned}$$

In particular, as a bounded quadratic form, the right side of (3.14) is weakly lower semicontinuous.

Now given a sequence $a_n\rightarrow 0$ and $\Psi _n \rightharpoonup \Psi$, we obtain from the bound above and weak lower semicontinuity

3.18 $$\begin{aligned}&\liminf _{n\rightarrow \infty } a_n^{-2\ell } F_{a_n}(\Psi _n) \nonumber \\&\quad \ge 4\pi \ell b_\ell \sum _{i \ne j}^N \int _{\mathbb {R}^{2(N-1)}} \mathrm e^{- \sum _{k, k\ne i,j }^N |x_k|^2} \chi _{i,R}(x_1,\dots , \, \not \! x_i, \dots , x_N) \nonumber \\&\qquad \times \int _0^\infty \mathrm e^{-r^2} \rho (r) \left| \frac{1}{2\pi \mathrm i} \oint _{|z_i-z_j|=r} \mathrm e^{-z_i \bar{z}_j }\frac{ \varphi (x_1,\dots ,x_N)}{(z_i-z_j)^{\ell +1}} dz_i \right| ^2 dr\, \prod _{j, j\ne i}^N dx_j \end{aligned}$$

where $\Psi (x_1,\dots ,x_N) = \mathrm e^{-\tfrac{1}{2} \sum _{i=1}^N |x_i|^2} \varphi (x_1,\dots ,x_N)$. Consider first the case when $\Psi \in \mathfrak {B}_\ell$. Then $\varphi$ is analytic and of the form $\varphi (x_1,\dots ,x_N) = \tilde{\varphi }(z_1,\dots ,z_N) \prod _{i<j} (z_i-z_j)^\ell$ for some analytic $\tilde{\varphi }$. Writing $\varphi (x_1,\dots ,x_N) = \xi _{ij}(z_1,\dots ,z_N) (z_i-z_j)^\ell$, we have

3.19 $$\begin{aligned} \frac{1}{2\pi \mathrm i} \oint _{|z_i-z_j|=r} \mathrm e^{-z_i \bar{z}_j }\frac{ \varphi (x_1,\dots ,x_N)}{(z_i-z_j)^{\ell +1}} dz_i = \mathrm e^{-|z_j|^2} \xi _{ij}(z_1,\dots ,z_j, \dots , z_j, \dots , z_N) \end{aligned}$$

in this case. Hence the right side of (3.18) equals

3.20 $$\begin{aligned}&4\pi \ell b_\ell \int _0^\infty \mathrm e^{-r^2} \rho (r) dr\sum _{i \ne j}^N \int _{\mathbb {R}^{2(N-1)}} \mathrm e^{- \sum _{k, k\ne i,j }^N |x_k|^2} \mathrm e^{-2 |z_j|^2}\chi _{i,R}(x_1,\dots , \, \not \! x_i, \dots , x_N) \nonumber \\&\qquad \quad \times |\xi _{ij}(z_1,\dots ,z_j, \dots , z_j, \dots , z_N)|^2 \prod _{j, j\ne i}^N dx_j . \end{aligned}$$

We have $\int \mathrm e^{-r^2} \rho (r) dr \ge \mathrm e^{-R^2}$, which goes to 1 as $R\rightarrow 0$. Moreover, by dominated convergence, we can replace $\chi _{i,R}$ by 1 in the limit $R\rightarrow 0$, and conclude that

3.21 $$\begin{aligned} \lim _{R\rightarrow 0} (3.20) = 4\pi \ell b_\ell \sum _{i\ne j} \langle \Psi | \mathfrak {D}^{(\ell )}_{ij} \Psi \rangle = 8\pi \ell b_\ell \, G^{(\ell )}(\Psi ) . \end{aligned}$$

Since $R>0$ was arbitrary, this yields the desired lower bound.

Next, consider the case when $\Psi \in \mathfrak {B}_{\ell '}$ for some $\ell ' < \ell$, but $\Psi \not \in \mathfrak {B}_{\ell '+1}$ (and hence, in particular, $\Psi \not \in \mathfrak {B}_\ell$). In this case, we can apply the bound (3.18) with $\ell$ replaced by $\ell '$, to conclude that $\liminf _{n\rightarrow \infty } a_n^{-2\ell } F_{a_n} (\Psi ) = + \infty$, as desired. Here we use the fact that the kernel of $\mathfrak {h}_{\ell '}$ equals $\mathfrak {B}_{\ell '+1}$, hence $\Psi$ is not in the kernel.

Finally, consider the case $\Psi \not \in \mathfrak {B}_0$. Then we can simply drop the interaction for a lower bound, and conclude that $\liminf _{n\rightarrow \infty } F_{a_n}(\Psi _n) \ge \Vert \sqrt{H^{(0)}} \Psi \Vert ^2>0$. In particular, $\liminf _{n\rightarrow \infty } a_n^{-2\ell } F_{a_n}(\Psi _n) = + \infty$ for any $\ell \ge 1$. This concludes the proof of the lower bound for $\ell \ge 1$.

### Upper Bound

We shall now prove part (2) of Prop. 1. Given the lower bound (3.3) we already established, we only need to prove (3.4) as an upper bound. It clearly suffices to consider the case $\Psi \in \mathfrak {B}_\ell$. In the opposite case $\Psi \not \in \mathfrak {B}_\ell$, the energy tends to infinity and we can simply take $\Psi _n = \Psi$ for all *n*, and use the lower bound (3.3).

For $\Psi \in \mathfrak {B}_\ell$, we consider the sequence

3.22 $$\begin{aligned} \Psi _n(x_1,\dots ,x_N) = \Psi (x_1,\dots ,x_N) \prod _{i<j} f(x_i - x_j) \end{aligned}$$

where $f (x) = f_{n,\ell }(x)=f_\ell (x/a_n)$, with $f_\ell$ the minimizer in (2.9). Since *f* is bounded and converges pointwise to 1 as $a_n\rightarrow 0$, $\Psi _n\rightarrow \Psi$ by dominated convergence. Using the representation (3.13), we have

3.23 $$\begin{aligned} F_{a_n}(\Psi _n) = \int _{\mathbb {R}^{2N}} |\Psi (x_1,\dots ,x_N)|^2 \left[ 4 \sum _{i=1}^N | \partial _{\bar{z}_i} S|^2 + \frac{1}{2}\sum _{i<j} v_{a_n}( |x_i-x_j |) | S |^2 \right] \prod _{j=1}^N dx_j \end{aligned}$$

for $S(x_1,\dots ,x_N) = \prod _{i<j} f(x_i-x_j)$. Since *S* is real, $| \partial _{\bar{z}_i} S| = \frac{1}{2} | \nabla _i S|$. Moreover, since $0\le f\le 1$, we can bound $|S|^2 \le f(x_i-x_j)^2$ for any pair $i\ne j$, as well as

3.24 $$\begin{aligned} \sum _{i=1}^N |\nabla S|^2 \le \sum _{i\ne j} |\nabla f(x_i-x_j)|^2 + \sum _{i\ne j\ne k} |\nabla f(x_i-x_j)| | \nabla f(x_k -x _j)| . \end{aligned}$$

We thus have to bound terms of the form

3.25 $$\begin{aligned} \mathrm{I}=\int _{\mathbb {R}^{2N}} |\Psi (x_1,\dots ,x_N)|^2\left\{ |\nabla f(x_i-x_j)|^2+\frac{1}{2}v_{a_n}(|x_i-x_j |)|f(x_i-x_j)|^2\right\} \prod _{l=1}^N dx_l \end{aligned}$$

and

3.26 $$\begin{aligned} \mathrm{II}=\int _{\mathbb {R}^{2N}}|\Psi (x_1,\dots ,x_N)|^2 |\nabla f(x_i-x_j)| | \nabla f(x_k -x _j)|\prod _{l=1}^N dx_l \end{aligned}$$

for $$i\ne j\ne k$$.

It follows from the definition (2.7) that as a quadratic form on $\mathfrak {B}_\ell$

3.27 $$\begin{aligned} \mathfrak {D}^{(\ell )}_{ij}= (\bar{z}_i-\bar{z}_j)^{-\ell } \mathfrak {D}^{(0)}_{ij}(z_i-z_j)^{-\ell }. \end{aligned}$$

Moreover, in the notation of [6],

3.28 $$\begin{aligned} \mathfrak {D}^{(0)}_{ij}=\delta _{ij}. \end{aligned}$$

Because of (3.27) and (3.28) we can rely on previous results proved in [6] for the case $\ell =0$. In fact, [6, Lemma 2] implies^5^ that for $\Psi \in \mathcal {B}_0$ and radial $g\ge 0$

3.29 $$\begin{aligned} \int _{\mathbb {R}^{2N}} |\Psi (x_1,\dots ,x_N)|^2\ g(x_i-x_j) \prod _{l=1}^N dx_l \le \int _{\mathbb {R}^2} g \, \langle \Psi | \delta _{ij} \Psi \rangle + C \int _{\mathbb {R}^2} g(x) \frac{|x|^4}{1+|x|^4} dx \, \Vert \Psi \Vert ^2 \end{aligned}$$

for some constant $C>0$, as well as

3.30 $$\begin{aligned} \int _{\mathbb {R}^{2N}}|\Psi (x_1,\dots ,x_N)|^2 g(x_i-x_j) g(x_k -x _j) \prod _{l=1}^N dx_l \le C \left( \int _{\mathbb {R}^2} g \right) ^2 \Vert \Psi \Vert ^2 \end{aligned}$$

for $$i\ne j\ne k$$.

We define

3.31 $$\begin{aligned} g(x)=|x|^{2\ell }(|\nabla f(x)|^2+ \tfrac{1}{2}v_{a_n}(|x|)|f(x)|^2) \end{aligned}$$

and write I as

3.32 $$\begin{aligned} \int _{\mathbb {R}^{2N}} |\Psi _{ij}(x_1,\dots ,x_N)|^2g(x_i-x_j)\prod _{k=1}^N dx_k \end{aligned}$$

where $\Psi _{ij}$ stands for $\Psi$ with a factor $(z_i-z_j)^\ell$ canceled. Note that by (3.27) we have

3.33 $$\begin{aligned} \langle \Psi _{ij}|\mathfrak {D}^{(0)}_{ij}\Psi _{ij}\rangle =\langle \Psi |\mathfrak {D}^{(\ell )}_{ij}\Psi \rangle \end{aligned}$$

and by (2.9)

3.34 $$\begin{aligned} \int _{\mathbb R^2} g =a_n^{2\ell }\, 4\pi \ell b_\ell . \end{aligned}$$

When applying (3.29), the first term gives after summation over *ij* the desired bound

3.35 $$\begin{aligned} a_n^{2\ell }\, 8\pi \ell b_\ell \,\langle \Psi | \mathfrak {h}_\ell \Psi \rangle . \end{aligned}$$

To bound the second term, we note that if *v* is supported in $\{|x|\le R_0\}$ then $v_{a_n}$ is supported in $\{|x|\le a_n R_0\}$. Pick an $R>R_0$ and split the integral in the last term in (3.29) into an integral over $\{|x|<a_nR\}$ and a remainder where $v_{a_n}=0$. The first part of the integral is bounded by $(\int g) R^4a_n^4$. For $|x|>a_nR$ we have $f(x)=(1-a_n^{2\ell }b_\ell /|x|^{2\ell })$ and thus $|\nabla f(x)|\sim a_n^{2\ell }/|x|^{2\ell +1}$ so $\int _{|x|>a_nR} |x|^{2\ell }|\nabla f(x)|^2 dx\sim a^{2\ell } R^{-2\ell }$. With $R=a_n^{-2/(\ell +2)}$ we see that the integral is smaller than $\int g$ by a factor $a_n^{4\ell /(\ell +2)}$. The whole term I therefore gives (3.35) as the leading contribution.

A bound on II can be obtained with the aid of (3.30). The resulting bound is of higher order than $\int _{\mathbb R^2} g$ (in fact, of the order $(\int _{\mathbb R^2} g)^2$) and vanishes upon multiplication by $a_n^{-2\ell }$ in the limit $a_n\rightarrow 0$. Altogether we thus obtain

3.36 $$\begin{aligned} \limsup _{n\rightarrow \infty } a_n^{-2\ell } F_{a_n}(\Psi _n) \le 4\pi \ell b_\ell \sum _{i\ne j} \langle \Psi | \mathfrak {D}^{(\ell )}_{ij} \Psi \rangle = 8\pi \ell b_\ell \, G^{(\ell )}(\Psi ). \end{aligned}$$

In combination with the lower bound, this concludes the proof of Theorem 1 for $\ell \ge 1$.

### The $\ell =0$ Case

The special feature of the $\ell =0$ case in two dimensions is that the minimizer $f_0$ of (2.11) depends on *R*, and the minimal value of (2.11) depends logarithmically on the parameters. By choosing *R* appropriately, the basic proof strategy goes through also for $\ell =0$, with only minor modifications compared to the case $\ell \ge 1$. Effectively, one is lead to the replacement

3.37 $$\begin{aligned} \ell b_\ell a^{2\ell }\rightarrow \frac{1}{\ln (R^2/a^2 b_0^2)} =\ln (1/a^2)^{-1}(1+o(1)) \end{aligned}$$

with *o*(1) tending to zero as $a\rightarrow 0$ for fixed *R*. With this replacement the Dyson Lemma in Eq. (3.5) holds also for $\ell =0$, and the estimates for the lower bound are obtained in the same way as for $\ell \ge 1$, by first letting $a\rightarrow 0$ followed by $R\rightarrow 0$.

For the upper bound the trial function *f* is defined as $f_0(r/a_n)$ where $f_0$ minimizes (2.11) with the choice $R= R'/a_n$ for some fixed $R'>0$ independent of *n*. In particular $\nabla f(r)=0$ for $r\ge R'$. Again letting $R'\rightarrow 0$ after $a_n\rightarrow 0$ (in order for the last term in (3.29) to be negligible compared to (3.34)), we conclude the upper bound

3.38 $$\begin{aligned} \ln (1/a_n^2)^{-1}(1+o(1))\, 8\pi \,\langle \Psi | \mathfrak {h}_0\Psi \rangle \end{aligned}$$

in place of (3.35).

## Extension to Three Dimensions

In this section we shall show analogous results in the three-dimensional case, with a strong confinement in the third direction. Consider a potential $V:\mathbb {R}\rightarrow \mathbb {R}$ such that the Schrödinger operator $-\partial _u^2 + V(u)$ has a ground state $\chi \in H^1(\mathbb {R})$ with corresponding energy *E*. On $L^2(\mathbb {R}^3)$, define $h\ge 0$ as

4.1 $$\begin{aligned} h = ( -\mathrm i\nabla + x \wedge e_3)^2 + V(x^{(3)}) - 2 - E \end{aligned}$$

where $e_3=(0,0,1)$ denotes the unit vector in the $x_3$-direction. For $v\ge 0$ radial and of compact support, we define

4.2 $$\begin{aligned} H_a = \sum _{i=1}^N h_i + \sum _{i<j} v_a ( | x_i-x_j | ) \end{aligned}$$

on $$\mathcal {H}= L^2(\mathbb {R}^{3N})$$, where $$v_a(x) = a^{-2} v( x /a)$$.

The relevant scattering parameters are now given by

4.3 $$\begin{aligned} b_\ell = \frac{1}{4 \pi (2\ell +1) } \min \left\{ \int _{\mathbb {R}^3} |x|^{2\ell } \left( |\nabla f(x)|^2 + \tfrac{1}{2} v(x) |f(x)|^2 \right) dx \, : \, \lim _{|x|\rightarrow \infty } f(x) = 1\right\} \end{aligned}$$

for $\ell \in \mathbb {N}\cup \{ 0\}$. The corresponding minimizers $f_\ell$ satisfy $0\le f_\ell \le 1$, and $f_\ell (x) = 1 - b_\ell /|x|^{2\ell +1}$ for $|x|> R_0$. In particular, $b_\ell = R_0^{2\ell +1}$ for hard spheres.

We introduce a sequence of closed subspaces

4.4 $$\begin{aligned} \mathcal {H}\supset \mathfrak {B}_0 \supset \mathfrak {B}_1 \supset \dots \end{aligned}$$

where $\mathfrak {B}_\ell$ for $\ell \ge 0$ consists of $\psi \in \mathcal {H}$ of the form

4.5 $$\begin{aligned} \Psi (x_1,\dots ,x_N) = \varphi (z_1,\dots ,z_N) \prod _{i<j} (z_i - z_j)^\ell \prod _{k=1}^N \chi (x^{(3)}_k) \mathrm e^{-\tfrac{1}{2} |z_k|^2 } \end{aligned}$$

with $\varphi : \mathbb {C}^N\rightarrow \mathbb {C}$ analytic. We write $x =(x^{(1)}_j, x^{(2)}_j, x^{(3)}_j)$ and identify $(x^{(1)}_j, x^{(2)}_j) \in \mathbb {R}^2$ with $z_j = x^{(1)}_j + \mathrm ix^{(2)}_j\in \mathbb {C}$. Note that again $\mathfrak {B}_0$ coincides with the kernel of $H^{(0)} = \sum _{i=1}^N h_i$, since for $\Psi (x) = \mathrm e^{- |z|^2/2 } \chi (x^{(3)}) \varphi (x)$,

4.6 $$\begin{aligned} \langle \Psi | h \Psi \rangle = \int _{\mathbb {R}^3} \mathrm e^{-|z|^2} |\chi (x^{(3)})|^2 \left( 4\left| \partial _{\bar{z}} \varphi (x) \right| ^2 + \left| \partial _{x^{(3)}} \varphi (x) \right| ^2 \right) dx. \end{aligned}$$

Obviously the spaces $\mathfrak {B}_\ell$ can be naturally identified with the corresponding spaces in two dimensions, by simply multiplying functions in the latter spaces by $\prod _{k=1}^N \chi (x^{(3)}_k)$. In particular, the operators $\mathfrak {D}^{(\ell )}$ naturally act on $\mathfrak {B}_\ell$ in the same way as in the two-dimensional case, and similarly for $\mathfrak {h}_\ell$.

Let $$c_0 = 1$$ and

4.7 $$\begin{aligned} c_\ell = \frac{\sqrt{\pi }}{2} \frac{\Gamma (1 + \ell )}{\Gamma (3/2 + \ell )} = \prod _{j=1}^\ell \frac{2j}{2j +1} \end{aligned}$$

for $\ell \ge 1$. Let also $\tilde{b}_\ell = b_\ell c_\ell$.

### Theorem 2

For any $\ell \ge 0$, $a^{-2\ell -1} H_a$ converges to $8\pi (2\ell + 1) \tilde{b}_\ell \int |\chi |^4 \, \mathfrak {h}_\ell$ as $a\rightarrow 0$ in the strong resolvent sense, i.e., for any $\mu > 0$ and $\Psi \in \mathcal {H}=L^2(\mathbb {R}^{3N})$

4.8 $$\begin{aligned} \lim _{a\rightarrow 0} \left( \mu + a^{-2\ell -1} H_a \right) ^{-1} \Psi = \left( \mu + 8\pi (2\ell +1) \tilde{b}_\ell \int |\chi |^4 \, \mathfrak {h}_\ell \right) ^{-1} P_\ell \Psi \end{aligned}$$

strongly in $L^2(\mathbb {R}^{3N})$, where $P_\ell$ denotes the projection onto $\mathfrak {B}_\ell \subset \mathcal {H}$.

The proof of Theorem 2 proceeds along very similar lines as the one in the two-dimensional case, hence we will not present it in full detail here. We merely point out the main differences.

The relevant pre-factor $(2\ell +1) \tilde{b}_\ell$ naturally arises through

4.9 $$\begin{aligned} 4 \pi (2\ell +1) \tilde{b}_\ell = \min \left\{ \int _{\mathbb {R}^3} |z|^{2\ell } \left( |\nabla f(x)|^2 + \frac{1}{2}v(x) |f(x)|^2 \right) dx \, : \, \lim _{|x|\rightarrow \infty } f(x) = 1\right\} \end{aligned}$$

with a factor $|z|^{2\ell }$ in place of $|x|^{2\ell }$ in (4.3). To see the validity of (4.9), note that for radial functions *f* we have

4.10 $$\begin{aligned} |z|^{-2\ell } \nabla |z|^{2\ell } \nabla f = |x|^{-2\ell } \nabla |x|^{2\ell } \nabla f \end{aligned}$$

hence the minimizer of (4.3) is also the minimizer of (4.9). The factor $c_\ell$ then results from averaging $|z|^{2\ell }$ over the unit sphere.

As for Theorem 1, Theorem 2 is proved by showing $\Gamma$-convergence of the corresponding quadratic forms. The upper bound follows in the same way as in two dimensions, using (4.9). Note that by definition

4.11 $$\begin{aligned} \int _{\mathbb {R}^{3N}} \frac{ |\Psi (x_1,\dots ,x_N)|^2}{|z_i-z_j|^{2\ell }} \delta (x_i-x_j) \prod _{k=1}^N dx_k = \int _\mathbb {R}|\chi |^4 \, \langle \Psi |\mathfrak {D}^{(\ell )}_{ij}\Psi \rangle \end{aligned}$$

for $\Psi \in \mathfrak {B}_\ell$, which explains the additional pre-factor $\int |\chi |^4$.

For the lower bound, the relevant Dyson Lemma reads as follows:

### Lemma 2

For any $\ell \ge 0$ and $R>a R_0$, and any $y = (\eta ,y^{(3)}) \in \mathbb {C}\times \mathbb {R}\equiv \mathbb {R}^3$, we have

4.12 $$\begin{aligned}&\int _{|x-y|< R} \mathrm e^{-|z|^2} | \chi (x^{(3)})|^2 \left( 4 |\partial _{\bar{z}} \varphi (x)|^2 + |\partial _{x^{(3)}} \varphi (x)|^2 + \tfrac{1}{2} v_a(|x-y|) |\varphi (x)|^2 \right) dx \nonumber \\&\quad \ge 4\pi (2 \ell +1) \tilde{b}_\ell a^{2\ell +1} \mathrm e^{|\eta |^2 -R^2 } \min _{|x^{(3)}|<R} |\chi (y^{(3)}-x^{(3)})|^2 \nonumber \\&\qquad \times \left| \frac{1}{4\pi c_\ell R^{2(\ell +1)} } \int _{|x-y|=R} \mathrm e^{-z \bar{\eta }} (\bar{z}-\bar{\eta })^{\ell } \varphi (x) d\sigma \right| ^2 \end{aligned}$$

where $\sigma$ denotes the surface measure on the sphere.

Its proof can be obtained by following the arguments in the two-dimensional case line by line. Note that if $\varphi$ is independent of $x^{(3)}$ and analytic in *z*, we have

4.13 $$\begin{aligned} \frac{1}{4\pi c_\ell R^{2(\ell +1)} } \int _{|x-y|=R} e^{-z \bar{\eta }} (\bar{z}-\bar{\eta })^{\ell } \varphi (z) d\sigma = \frac{1}{2\pi \mathrm i} \oint _{|z-\eta |=R} e^{-z \bar{\eta }}\frac{ \varphi (z)}{(z-\eta )^{\ell +1}} dz \end{aligned}$$

where *dz* is again the complex line element. The right side is independent of *R* in this case, and coincides with the corresponding expression in two dimensions. Using that, as an $H^1$-function, $\chi$ is uniformly Hölder continuous, one easily sees that

4.14 $$\begin{aligned} \lim _{R\rightarrow 0} \int _\mathbb {R}|\chi (t)|^2 \min _{|x|<R} |\chi (t-x)|^2 dt = \int _\mathbb {R}|\chi |^4. \end{aligned}$$

The remainder of the proof of the lower bound then proceeds as in Sect. 3.3.

Assuming the potential *V* to be confining, i.e., $\lim _{t\rightarrow \pm \infty } V(t) = + \infty$, and adding an additional confining potential $\lambda \sum _{i=1}^N |z_i|^2$ in the perpendicular directions, the analogue of Corollary 1 can be seen to hold in the three-dimensional case as well. The relevant Laughlin wavefunctions are simply given by (1.1) multiplied by $\prod _{k=1}^N \chi (x_k^{(3)})$, the ground state in the direction of the magnetic field.

## Footnotes

1 A standard reference on the quantum Hall effect is [2]; see also the reviews [3] and [4].

2 The term “pseudo-potentials” is often used for the expansion coefficients in (1.5) rather than the projection operators (1.2).

3 The magnetic length for a charge *e* in a field of strength *B* is $\sqrt{\hbar /eB}$. In our units $\hbar =e=1$ and $B=2$, so the magnetic length is $1/\sqrt{2}$.

4 Alternatively, we could consider the system for a fixed *v* (corresponding to the choice $a=1$) and subject to a magnetic field $B>0$. Via a rescaling of lengths, the resulting Hamiltonian is then unitarily equivalent to *B*/2 times $H_{\sqrt{B/2}}$. In other words, the parameter *a* in (2.2) has the physical interpretation of the ratio of the scattering length of the interaction potential to the magnetic length.

5 Lemma 2 in [6] is stated in three dimension, but an inspection of its proof shows that it equally holds in the two-dimensional case as well.
